# How great is current curative expenditure and catastrophic health expenditure among patients with cancer in China? A research based on “System of Health Account 2011”

**DOI:** 10.1002/cam4.1590

**Published:** 2018-06-20

**Authors:** Ang Zheng, Wenjuan Duan, Lin Zhang, Xintong Bao, Xiaoyun Mao, Zhuojun Luo, Feng Jin

**Affiliations:** ^1^ Department of Breast Surgery The First Affiliated Hospital of China Medical University Shenyang Liaoning Province China; ^2^ Department of Social Medicine and Health Management China Medical University Shenyang Liaoning Province China; ^3^ Seven‐year System of Clinical Medicine Education China Medical University Shenyang Liaoning Province China; ^4^ Departments of Surgical Oncology and Breast Surgery The First Affiliated Hospital of China Medical University Shenyang Liaoning Province China

**Keywords:** cancer, catastrophic health expenditure, current curative expenditure, influencing factors, SHA 2011

## Abstract

In recent years, the incidence and mortality of cancer have witnessed a dramatic increase. Cancer has already caused severe economic burdens on society, especially in developing countries and has become a major public health concern. This study evaluates the medical economic burden, including total current curative expenditure (CCE) and catastrophic health expenditure (CHE) on cancer in Liaoning Province, China. A total of 252 medical institutions were investigated with multistage stratified cluster random sampling. We established a standardized database of 3 532 517 samples. “System of Health Account 2011”, a new internationally recognized accounting system, was established to analyze the CCE on six most common cancers. CHE were estimated from the extracted 1344 patients with cancer, which performed a cross‐sectional study. The association of individual and contextual factors with CHE was evaluated using logistic regression models. CCE for all the patients with the six types of cancer was 2801.38 million CNY in Liaoning Province, the highest of which was lung cancer. The incidence of CHE was 42.78%, while the threshold was 40%. The average and relative distance were 10.41% and 24.32%, respectively. Influencing factors were length of stay, type of health insurance, location of household, etc. Our findings highlight the need to address medical economic burden in the cancer population. Households with the cancer are more likely to incur CHE. Financial intervention to prevent it should target on poor households. We provide suggestions in aspects of health insurance and health service management to reduce CHE.

## 1 INTRODUCTION

According to the calculation of WHO in 2011, cancer resulted more in deaths than cardiovascular and cerebrovascular diseases.^1^ At the same time, the ongoing global population and epidemiological transformation can lead to ever‐growing disease burdens on low‐ and middle‐income countries for the following decades. Indeed, it was estimated that, by 2025, there would be 20 million more new cases of cancer each year all over the world.^2^ In health system, cancer has already caused onerous burdens on both developed and developing countries.^3^ In China, a latest research showed that the gross incidence of cancer was about 284.55/100 000 in 2013. Among all new cases, lung cancer ranked the first, followed by gastric cancer, liver cancer, colon cancer, and esophageal cancer. The incidence rate of cancer in Liaoning Province of China was 331.9/100 000, higher than the national level. The incidence rates of malignant tumor among males and females were 345.1/100 000 and 319.5/100 000,^4^ respectively. Accounting for a high burden of morbidity and mortality in Liaoning Province, such kinds of cancers can give rise to catastrophic health expenditure and impose a substantial financial burden.

Studies on CHE and its influencing factors have been carried out globally, using microdata to evaluate financial burdens of residents. A retrospective observational study in 133 countries illustrated that CHE whose threshold supposed to be 10% rose from 9.7% to 11.7% in just 5 years’ time from 2005 to 2010.^5^ Another study on CHE conducted in 59 countries found that as the proportion of household health expenditure to total health expenditure increased by 1%, the incidence of CHE would correspondingly increase by 2.2%.^6^ Additionally, limited studies assessed the CHE of population with a specific disease, such as cancer. A study conducted in South Korea by Choi et al^7^ showed that 39.8% of the 211 cancer patients suffered from CHE. While in South Asia, 48% of the 9513 patients with cancer experienced CHE.^8^ It also showed that the incidence of CHE varied among different countries. In China, with relatively high proportion of out‐of‐pocket (OOP) and the complexity and severity of cancer, the expenditure on its diagnosis and treatment in general can be higher than that on other diseases, which leads to countless incidents of CHE. However, hardly any study assessed the CHE and its influencing factors of cancer in China.

Based on present knowledge, annual medical expenditure of cancer patients in China was about 80 billion CNY, constituting about 20% of total cost of health, which was much higher than the cost of other chronic diseases, and was regarded as an essential factor contributing to the rising of total health expenditure.^9^ In this case, we collected expenditure information of cancer patients in Liaoning and used “System of Health Account 2011” (SHA 2011) to count total current curative expenditure.SHA 2011 is a new health care accounts system, which keeps the former triaxial relationship and develops three analytical interfaces in order to fix the existing shortcomings and make it more convenient for analysis and comparison across countries. SHA 2011 was introduced into China in 2014 and few about its application in China has been reported. In SHA 2011, the current health expenditure is used instead of the original health care expenditure. Current health expenditure, different from health care expenditure, refers to the expenditure of direct treatment that does not include the expenditure of fixed assets. The costs directly calculated by current health expenditure are referred to as current curative expenditure.^10^, ^11^ Notably, this study put special emphasis on CHE resulted by gastric cancer, liver cancer, lung cancer, esophageal cancer, breast cancer, and colon cancer, which were the major cancers bringing huge disease burdens on Chinese urban population.^12^ Because of lacking studies on such research, a study on CHE could efficiently decrease cancer expenditure, avoid withdrawing treatments, and better protect the right to health and life, as well as achieve Universal Health Coverage proposed by WHO.

Generally speaking, CHE is measured in two methods. One is OOP health care expenditure as a percentage of total household consumption expenditure. When the ratio exceeds a certain value, it is considered to incur CHE. A threshold of 10% is generally accepted in academic circles.^13^The other is OOP health care expenditure as a percentage of household nonfood expenditure. It is defined as ‘catastrophic’ if the ratio exceeds 40%, which is the accepted threshold.^14^ The essential difference between these two approaches is that the latter excludes the rigid impact of household food expenditure and avoid the deviation of the catastrophic health expenditure measurement process in low‐income households. Therefore, in this study, OOP health care expenditure as 40% of household nonfood expenditure was studied,^15^ and 30% and 50% threshold were set for comparison. The incidence of CHE reflects the density and depth of CHE among households.^16^

## 2 METHODS

### 2.1 Data sources

Macrodata used in this study were extracted from the 2015 Liaoning Health Statistical Yearbook (Bureau of statistics of Liaoning Province), 2015 Liaoning Health Financial Yearbook (Liaoning province finance department), China National Health Accounts Report 2015 (China National Health Development Research Center), and Liaoning Health Accounts Report 2015 (Health and Family Planning Commission of Liaoning Province).

### 2.2 Study sample

In this study, multistage stratified cluster random sampling was used. The first stage was to choose sample cities from Liaoning province based on full consideration into the perfection of health information management system and level of economic development. The second stage was to select one county and one district in each city. Then three township hospitals were extracted from every country and three community health service organizations were extracted from every district. Three village clinics and individual clinics were selected in rural town or community. The third stage was to choose medical institutions and public health institutions according to type of institutions or administration structure. A total of 252 medical institutions and specialized public health institutions, among which 7 were provincial hospital, 58 institutions in Dalian, 62 institutions in Liaoyang, 64 institutions in Panjin, and 61 institutions in Tieling. The basic information was collected from institute information system (including data of outpatient and inpatient care), including gender, age, season, disease, expense, and region, length of stay. Data gathered were then classified and coded according to the *International Classification of Disease, Tenth Revision* (*ICD‐10*), collecting 3 532 517 valid subjects after removing the incomplete and void data. A standardized basic database was established.

Questionnaire surveys were carried out among the patients, which collected information of demographic characteristics, household income and expenditure, medical expenses and compensation. Fifteen hundred questionnaires were distributed in sample cities. Ultimately, 1344 were completed and standards compliant, with 89.60% effective rate.

### 2.3 Calculating CCE for six types of cancer accounted based on SHA 2011

Gastric cancer, liver cancer, lung cancer, esophageal cancer, breast cancer, and colon cancer are each coded as C16.9, C22.9, C34.9, C15.9, C50.9, and C18.9 according to *ICD‐10*. The costs of cancer treatment include medical service expenditure, government subsidies, and basic expenditure subsidies are from *2015 Liaoning Health Statistical Yearbook and 2015 Liaoning Health Financial Yearbook*. Medical service income consists of expenditure from treating outpatient and inpatient, and health statistics can be used for total quantity control. For project expenditure subsidies, it can be obtained from 2015 Liaoning Health Financial Yearbook. The calculation of basic expenditure subsidies uses the allocation principles of equivalent person‐time, allocating the subsidies to treatment service and prevention service.

Treatment service expenditure data from subjects need to be broken down into different disease categories and different age groups. Firstly, basic expenditure subsidies were broken down into outpatient service category and inpatient service category in terms of the workload proportion of each. For outpatient service category, we allocated the subsidies to different diseases according to numbers of outpatients of each disease and then the subsidies of each disease were broken down into different age groups. The same conduct was carried out for inpatient category except that this category was arranged according to the length of stay to allocate subsidies to different diseases. Secondly, project expenditure subsidies were allocated to the target diseases and the target population according to the treatment requirement of the project. Thirdly, service incomes were broken down into groups according to age and the costs of treating different disease of outpatients and inpatients.

### 2.4 Calculating CHE of cancer

#### 2.4.1 Incidence and intensity of household CHE

Since food is a necessity good, we used nonfood expenditure as the denominator of measurement for CHE so as to exclude bias which may occur when measuring CHE in poor households and to assess OOP as a percentage of household income net of subsistence needs (calculated as total household income minus the cost for subsistence needs, eg food). In the equation below, *E*_*i*_ indicates whether CHE has occurred. The formula is shown as follows:

(1) $$E_{i}=\left\{\begin{array}{cc} 0 & ifT_{i}/\left(x_{i}-f\left(x\right)\right)<z\\ 1 & ifT_{i}/\left(x_{i}-f\left(x\right)\right)\geq z \end{array}\right.$$

*T*_*i*_ is the annual household OOP for health, *x*_*i*_ is the annual household consumption expenditure, *f*(*x*) is the household expenditure on food, and *z* is the set threshold. The incidence of CHE referred to households suffering from CHE to as a percentage of total households. We could calculate the incidence of CHE in households with cancer patients according to *E*_*i*_, and in order to estimate the intensity of it, mean catastrophic payment gap and mean positive gap were introduced. The equations below:

(2) $$H=\frac{1}{N}\sum_{i=1}^{N}E_{i}$$

(3) $$O=\frac{1}{N}\sum_{i=1}^{N}E_{i}\left(\left(T_{i}/x_{i}\right)-z\right)$$

(4) $$\text{MPO}=\frac{O}{H}$$

*H* is the incidence of CHE, *O* is the average distance, and MPO is the mean relative distance. The former reflects the intensity of CHE in the overall households with cancer patients, and the latter indicates the intensity of CHE in the households suffering from CHE.

#### 2.4.2 Influencing factors of CHE among households

In the study, we chose 40% as the threshold of CHE, based on logistic regression analysis employed to analyze factors that affect CHE including age, sex, and level of education (Table 1). The formula is shown below:

**Table 1:** Demographic, socioeconomic characteristics of study population, and variable assignment

Characteristic	n	%	Variable assignment
Age			
<40	39	2.9	1
40‐60	551	41.0	2
60‐80	645	48.1	3
≥80	109	8.1	4
Gender			
Female	569	42.4	1
Male	775	57.6	2
Level of education			
0‐6 y (Primary)	310	23.1	1
7‐12 y (Secondary)	878	65.3	2
>12 y (Tertiary)	156	11.6	3
Financial support			
Yes	485	36.1	1
No	859	63.9	0
Outpatient services			
Yes	683	50.8	1
No	661	49.2	0
The proportion of out‐of‐pocket on total health expenditure (%)			
<30	16	1.2	1
30‐60	552	41.1	2
≥60	776	57.7	3
Length of stay			
<5	205	15.2	1
5‐10	398	29.6	2
10‐15	248	18.4	3
15‐20	206	15.3	4
≥20	287	21.3	5
Household size			
<3 people	408	30.3	1
3‐5 people	392	29.1	2
5‐7 people	288	21.4	3
≥7 people	256	19.1	4
Household income level			
<5000	67	5.1	1
5000‐10 000	64	4.8	2
10 000‐30 000	260	19.3	3
30 000‐50 000	401	29.8	4
≥50 000	552	41.0	5
Location of household			
Urban	820	61.0	1
Rural	524	39.0	0
Type of health institutions			
Tertiary hospital	733	54.5	1
Not tertiary hospital	611	45.5	0
Household with at least one person ≥65‐year‐old			
Yes	453	33.7	1
No	891	66.2	0
Type of health insurance			
Urban employee basic medical insurance	580	43.1	1
Urban resident basic medical insurance	226	16.8	2
New cooperative medical scheme	337	25.1	3
Others	201	14.9	4

(5) $$y=\alpha +\sum \beta_{i}X_{i}+\varepsilon$$

*y* is the dependent variable, α is the constant, *X*_*i*_ is the independent variable, and β_*i*_ is the regression coefficient of *X*_*i*_. The dependent variable indicates whether or not CHE occurred (1 for CHE, 0 for no CHE).

All statistical analyses were performed with SPSS software, version 22.0, and STATA software, version 12.0.

## 3 RESULTS

### 3.1 Calculating CCE for six types of cancer

Based on “SHA 2011”, the results showed the CCE for cancers (gastric cancer, liver cancer, lung cancer, esophageal cancer, breast cancer, and colon cancer) were 2801.38 million CNY, and the CCE for lung cancer was the highest, up to 928.8 million CNY (1 USD≈8.27 CNY, 2014), followed by gastric cancer (567.18 million CNY), breast cancer (555.784 million CNY), liver cancer (320.8584 million CNY), colon cancer (216.5018 CNY), and esophageal cancer (212.26 million CNY).

**Figure 1:**
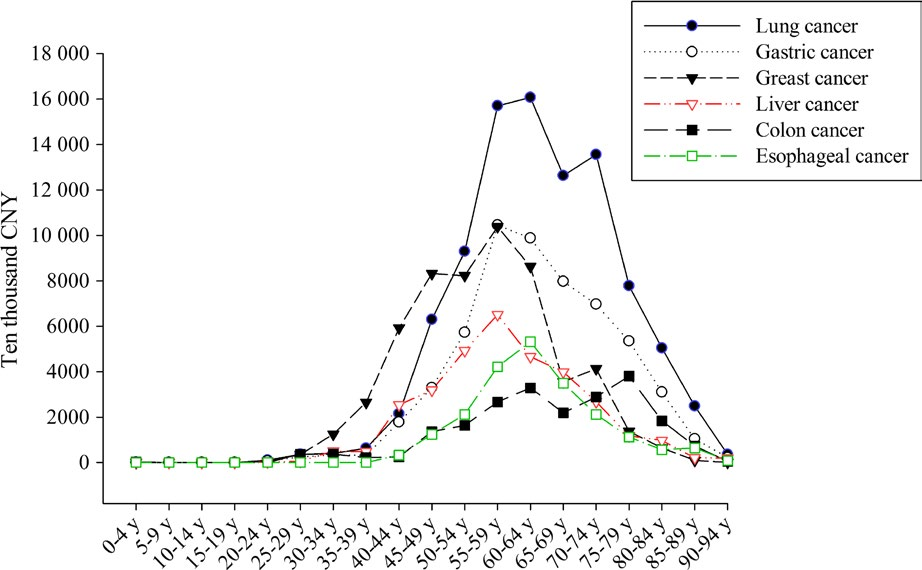
The CCE of cancer distribution in different age group

As for CCE by age, a significant part of the expenditure occurred in groups aged 50‐70 y, accounting for nearly 70% of six types of cancer. However, in terms of CCE, it varied in different cancers. The highest CCE of cancer in this study were mainly in the 50‐60 y, while breast cancer showed a different tendency with expenditure occurring mainly in younger groups aged 45‐64 (Figure 1).

### 3.2 CHE among cancer patients

#### 3.2.1 The incidence of CHE among cancer patients

The incidence was 46.27%, 42.78%, and 32.14%, respectively, when the threshold of CHE was 30%, 40%, and 50%. The lower the threshold was, the higher the incidence would be. Among the households, those with esophageal cancer patients were most likely to experience CHE of which the incidences were 60.29%, 57.89%, and 46.89% measuring with different thresholds. The incidence of CHE among households with breast cancer patients was the lowest, following lung cancer, gastric cancer, liver cancer, and colon cancer, which were sorted by incidence in descending order.

#### 3.2.2 The intensity of CHE

Average distance and relative disparity of CHE decreased when threshold varied from 30% to 50%. At a given threshold, the intensities of CHE in descending order were esophageal cancer, lung cancer, gastric cancer, liver cancer, colon cancer, and breast cancer. As recommended by Xu et al,^6^ we adopted 40% as the threshold. The incidence of CHE among sample households with the six types of cancer was 42.78% and the average distance was 10.41%. It indicated that among the sample households, when the ration of OOP to household income net of food spending exceeded 40% of threshold, the mean average intensity was 10.41%. In this case, the relative distance was 24.32%,suggesting that households who have suffered from CHE spent an average of 64.32% of their household incomes net of food spending on OOP. For each type of cancer, households spent 72.99% (esophageal cancer), 68.61% (colon cancer), 62.69% (lung cancer), 60.19% (gastric cancer), 58.04% (breast cancer), and 57.98% (liver cancer) of their household incomes net of food spending on OOP on average (Table 2).

**Table 2:** The incidence and intensity of CHE among households with each type of cancer patients

	Threshold		
	30%	40%	50%
Six types of cancer			
Incidence (%)	46.27	42.78	32.14
Average distance (%)	14.81	10.41	6.61
Relative distance (%)	32.01	24.32	20.45
Esophageal cancer			
Incidence (%)	60.29	57.89	46.89
Average distance (%)	25.01	19.10	13.89
Relative distance (%)	41.48	32.99	29.62
Colon cancer			
Incidence (%)	57.08	52.05	39.26
Average distance (%)	20.29	14.89	10.01
Relative distance (%)	35.55	28.61	26.36
Gastric cancer			
Incidence (%)	44.26	39.63	24.88
Average distance (%)	12.12	8.00	4.76
Relative distance (%)	27.39	20.19	19.13
Liver cancer			
Incidence (%)	43.88	40.09	29.25
Average distance (%)	11.37	7.21	3.63
Relative distance (%)	25.91	17.98	12.42
Lung cancer			
Incidence (%)	40.95	38.79	33.19
Average distance (%)	12.75	8.80	5.17
Relative distance (%)	31.13	22.69	15.59
Breast cancer			
Incidence (%)	34.12	30.98	22.75
Average distance (%)	8.79	5.59	2.83
Relative distance (%)	25.77	18.04	12.48

### 3.3 Influencing factors of CHE among households

The results showed that these factors were length of stay, type of health insurance, household size, etc. As we can see from the results, increased length of stay, no insurance, low level of education, more people in family, high OOP%, household with at least one old person over 65 years of age and low‐income level, were more likely to lead to CHE among households with cancer patients. On the other hand, age, sex, etc. were not significant influencing factors in CHE.(Tables 1 and 3).

**Table 3:** Results of logistic regression of influencing factors of CHE among households

Variable	*B*	SE	Wald	*P*‐value	OR	95% CI	
						Lower	Upper
Length of stay	0.63	0.06	116.62	.00	1.88	1.68	2.11
Type of health insurance	0.16	0.07	4.92	.03	1.18	1.02	1.36
Household size	0.16	0.07	5.52	.02	1.17	1.03	1.33
The proportion of out‐of‐pocket on total health expenditure (OOP %)	1.55	0.16	98.23	.00	4.70	3.46	6.39
Type of health institutions	1.04	0.15	46.94	.00	2.83	2.10	3.82
Lever of education	−1.94	0.16	156.41	.00	0.14	0.11	0.19
Household with at least one person ≥65‐year‐old	0.59	0.17	12.33	.00	0.56	0.40	0.77
Household income level	−0.58	0.08	53.15	.00	0.56	0.48	0.65
Constant	−1.36	0.74	3.39	.07	0.26		

OR, Odds Ratio; 95% CI, 95% Confidence Interval.

## 4 DISCUSSION

### 4.1 The CCE of cancer

The study found that the total CCE of six types of cancer reached 2801.38 million CNY, accounting for 3.63% of the total expenditure on health, 0.098% of gross regional domestic product, and 0.55% of public financial budget expenditure.^17^ Not only in Liaoning Province, the total expenditure on cancer treatment was also very high in the whole country in 2008, with up to more than 100 billion CNY (1 USD≈6.94 CNY, 2008).^18^ In other countries, the expenditure on cancer treatment was also high—the financial burden of cancer in South Korea in 2005 was 14.1 million dollars.^19^ While our study found that health expenditures on lung cancer, gastric cancer, and breast cancer were the top three among the six types of cancer, accounting for 73.24% of the total costs. Study conducted in more developed regions in China indicates that the per capita medical cost among lung cancer patients was up to 19 922.24 CNY in 2014, accounting for most of urban residents’ income and far exceeding the disposable income of rural residents.^20^

The CCE of cancers were also different in various age groups. This study showed that the disease burden of cancer was mainly on people aged from 50 to 70. Vesna Zadnik et al found that the main financial burden—which increased with age—was on the elderly population and for both male and female, cancer was predominant in people aged from 50 to 74,^21^ presenting results similar to ours. However, for cancer control in the future, cancer prevention and control measures cannot be carried out only among the elderly population.

### 4.2 CHE among households with cancer patients

The overall incidence of CHE for Chinese households is 4.81% (threshold of 40%) and 12.61% (threshold of 10%).^22^ The incidence of CHE among households with cancer patients in Liaoning Province was 42.78%. In the study conducted in the rural area in Shandong Province, the incidence was about 31.43%, lower than the incidence we estimated in our study. The research led by Sujin also indicated that people living in less developed regions were more likely to suffer CHE compared with those in developed regions.^23^ Therefore, expanding health and welfare policy is necessary, which will enable poor people receive more financial supports, so that low‐income households with cancer patients can get protections against CHE. Studies showed that after implementing better health policy, low‐income cancer patients would have a higher utilization rate for health care services.^24^ As for other countries, family members with liver cancer could possibly led to CHE in South Korea. The incidence of CHE among households with cancer patients in South Korea was 39.8%, lower than that of CHE in China.^7^ Besides CHE among cancer patients, a study in Vietnam showed that the number of households with CHE and impoverishment increased during the period of 2002‐2010.^25^ In Nepal, about 14% households were faced with CHE.^26^

### 4.3 Influencing factors of CHE among households with cancer patients

In this study, factors significantly affecting CHE among households with cancer patients were length of stay, type of health insurance, household size, etc. As we can see from the results, increased length of stay, no insurance, low level of education, more people in family, high OOP%, household with at least one person over 65 years of age and low‐income level, these factors had major impact on CHE. When CHE was defined as OOP medical costs exceeding 30% of annual household income by some researchers, it was found that education background, income level, type of health institutions, and health insurance were influencing factors of CHE in the study on influencing factors of CHE among households with cancer in 10 countries in southeast Asia, of which the results are similar to ours.^8^ Also, the study found that cancer stage was one of the factors—the further the stage extended, the more likely CHE would occur. The study conducted in Vietnam on influencing factors of CHE among urban residents found that having a member who aged 65 and above in the family could have significant influence on CHE.

Besides those studies mentioned above, another study found that the families with female as head of household and job changes were more prone to CHE and family size was also a influencing factor.^7^ But in our study, family size was not one of the influencing factors of CHE among households with cancer patients. This might be explained by the unique family structure in China. The productive forces in a family were husband and wife, and the old and the young had no sources of income. Generally, a family in China was made up of the old, a married couple, and a child. Therefore, family size was not included in the influencing factors.

### 4.4 Suggestions to policy‐makers

Up to now, the coverage of health insurance is high in China, but the depth is limited and needs improving. Also, health insurance and welfare package does not put its focus on medically and financially fragile population with cancer.^27^ As a result, future optimization and adjustment should be implemented, putting more emphasis on the old and fragile population. Specifically, the proportion of health care compensation for cancer patients should be further improved to reduce risks of CHE among households and reduce the tendency that the poor are more susceptible to CHE. Based on the differences between rural and urban areas, the authorities should further improve the reimbursement ratio of hospitalization costs in health institutions of all levels, and continue to improve the mechanism of outpatient care compensation of cancer and increase the actual compensation ratio.

With the increase of income level and the awareness of residents’ health care, the standard of consumption demand for medical treatment has been improved. Many patients choose large hospitals. This situation is also related to limited capacity of medical care in primary hospitals. Therefore, it is suggested that a community‐based service system should be established to meet the needs of residents for health services and regular physical examinations should be organized, especially for elderly people.

Strict control in cost such as shortening hospitalization time and reducing clinic visits, can reduce economic loss of patients. Measures such as improving quality of medical care, strengthening doctor's occupational morals and technique skills, and improving diagnostic accuracy, are preferable in the control progress.

## PATIENT CONSENT

All the participants agreed our investigation request and each participant signed on their consents which were recorded and kept properly by the Health Economics Association of Liaoning Province.

## ETHICAL APPROVAL

The study was supported by Health Economics Association of Liaoning Province and Ethics Committee of China Medical University and they claimed that they approved this study. All procedures performed in studies involving human participants were in accordance with the ethical standards of the institutional and national research committee and with the Helsinki declaration and its later amendments or comparable ethical standards. All the informed consent form and the data we used have been informed to Ethics Committee of China Medical University and got their permission.

## CONFLICT OF INTEREST

The authors have declared that no competing interests exist.
